# Environmental Change Can Result in Irreversible Biodiversity Loss in Recently Formed Species Flocks

**DOI:** 10.1111/gcb.70239

**Published:** 2025-05-10

**Authors:** Hanna ten Brink

**Affiliations:** ^1^ Institute for Biodiversity and Ecosystem Dynamics (IBED) University of Amsterdam Amsterdam the Netherlands; ^2^ Department of Fish Ecology and Evolution, Center of Ecology, Evolution, and Biogeochemistry Eawag Swiss Federal Institute of Aquatic Science and Technology Kastanienbaum Switzerland

**Keywords:** adaptive radiation, biodiversity, climate change, coexistence, environmental change, ontogenetic diet shifts, regime shifts, size structure

## Abstract

Adaptive radiations, where a lineage diversifies into multiple species exploiting different niches, are key drivers of biodiversity. It is therefore important to understand the factors that drive such radiations and how changing environmental conditions affect their persistence. Using a size‐structured model, I study how changing environmental conditions impact the persistence of a six‐species flock. At birth, individuals are constrained to feed on a shared resource. As they mature, individuals diversify into six specialized forms, each adapted to feed on specific resources. Environmental changes affecting one species can trigger a cascade, altering the size structure of the focal species and subsequently affecting resource availability for other species. Under these altered ecological conditions, coexistence of all species becomes impossible. Importantly, once species are lost, they cannot re‐establish even when environmental conditions return to their original state, resulting in irreversible biodiversity loss. These findings underscore the vulnerability of species flocks to environmental change and highlight the potential for unexpected outcomes in the face of shifting ecological conditions due to climate change.

## Graphical abstract

Environmental changes affecting a single species can trigger cascading effects resulting in the extinction of other species. This study shows how shifts in the size structure of one species can increase competition among small juveniles of multiple species, leading to irreversible biodiversity loss. These results reveal a new mechanism behind sudden ecosystem collapses and highlight the potential for unexpected outcomes in the face of shifting ecological conditions.

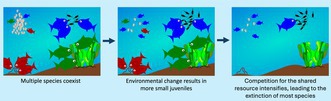

## 1 Introduction

Adaptive radiation has played a crucial role in shaping much of the biodiversity we observe today (Schluter 2000b). It refers to the rapid evolutionary divergence of members of a single ancestral species into multiple adaptive forms, each exploiting different ecological niches. Most examples of adaptive radiation are found on islands or in lakes, environments that are relatively rich in ecological opportunity, a prerequisite for adaptive radiation (Gillespie et al. 2020; Stroud and Losos 2016).

The endemic nature of many adaptive radiations makes these species highly susceptible to extinction driven by environmental change. Recently diverged sympatric species often lack strong intrinsic postzygotic isolation, so shifts in the selection regime due to environmental change can increase gene flow, leading to speciation reversal and the erosion of genetic and ecological differences (Seehausen et al. 2008). Alternatively, environmental change can alter both the biotic and abiotic conditions of habitats and thereby undermine the conditions necessary for the coexistence of endemic species flocks. While species reversal as a driver of diversity loss has been well studied (e.g., Alexander et al. 2017; Bhat et al. 2014; Gilman and Behm 2011; Vonlanthen et al. 2012), less attention has been given to how environmental change can disrupt the conditions necessary for the coexistence of recently formed species flocks. The aim of the current study is to address this knowledge gap by studying how environmental change can alter coexistence dynamics.

Coexistence of closely related species within adaptive radiations is strongly influenced by the strength of interspecific competition (Germain et al. 2020). When species compete for similar resources, competitive exclusion can occur, where one species outcompetes and displaces the other. However, in adaptive radiations, interspecific competition often drives ecological specialization, as species evolve to exploit different niches, reducing competition (Schluter 2000a). This process of niche partitioning allows for the coexistence of multiple species within the same environment and promotes the diversification characteristic of adaptive radiations (Dieckmann and Doebeli 1999; Gillespie et al. 2020).

Coexistence of endemic species flocks is further complicated by ontogenetic diet shifts, where resource use changes with body size throughout an individual's life. Classical theory predicts that multiple competitors can coexist if they partition resources differently (Schoener 1974). However, this theory assumes that all individuals within a consumer species affect resources equally, ignoring the fact that many species grow in body size during their life (Werner and Gilliam 1984), often accompanied by changes in resource use. Such ontogenetic diet shifts are widespread in nature (Werner 1988; Werner and Gilliam 1984) and influence the conditions for coexistence by altering where in the life cycle density‐dependent regulation occurs, which determines whether species can persist together (Schellekens et al. 2010). Because of their small size, newborn individuals are often constrained in the type of food they can eat (Werner 1988). Therefore, in many taxa, diets are similar during the early life stages, and only later in life do juveniles and adults diverge into a broad spectrum of feeding strategies (Brickle et al. 2003; Nunn et al. 2012; Sánchez‐Hernández et al. 2019; Werner and Gilliam 1984; Wilbur 1980). This pattern is also observed in recently formed species flocks (Chouinard and Bernatchez 1998; Damerau et al. 2012; van Zwieten et al. 2016; Wellenreuther and Clements 2008; Wood et al. 1999). For example, large cichlid fry are nearly all planktivorous, irrespective of the highly diverse adult feeding specialization (van Zwieten et al. 2016). Another example is the lake whitefish ( *Coregonus clupeaformis* ) species complex in North America, where sympatric pairs of ecotypes show distinct specializations as adults. However, during the larval stage, there is no difference in diet between the two ecotypes (Chouinard and Bernatchez 1998). Such shared dependence on similar resources during early life stages has important implications for the conditions for speciation, potentially limiting adaptive radiation when competition for shared resources among small individuals is strong (ten Brink and Seehausen 2022).

Environmental change can disrupt species interactions, potentially destabilizing coexistence and altering community structure. For instance, Jiang and Morin (2004) demonstrated that two ciliate species coexisted at low and high temperatures, while competitive exclusion occurred at intermediate temperatures, likely due to a change in metabolic requirements. This illustrates how changing environmental conditions can alter species interactions, a concern that is particularly relevant for endemic species flocks, which often have narrow ecological niches and limited dispersal ability. Understanding how disruptions impact species coexistence is especially urgent in the current era of climate change, as ecosystems undergo rapid shifts in climatic conditions affecting many biological processes such as physiology, phenology, and population dynamics (Scheffers et al. 2016), leading to biodiversity loss (Thomas et al. 2004) and restructuring of food webs (e.g., Gauzens et al. 2024; Lindmark et al. 2019).

While environmental change can include multiple factors, such as habitat loss, pollution, and the introduction of invasive species, the current study focuses on changes in food productivity and mortality regime. Food availability determines individual‐level processes such as growth and reproduction, directly affecting birth rates, while mortality determines survival rates. Climate change can impact both factors, for instance, by altering food availability through shifts in prey distributions (e.g., Carter et al. 2017) or primary production (e.g., Moore et al. 2018), and by modifying mortality regimes through species‐ or size‐specific effects, such as increased temperature stress (e.g., Dahlke et al. 2020; Pörtner and Knust 2007) or altered predator–prey interactions (e.g., Parmesan 2006; Renner and Zohner 2018). Although many factors may influence species persistence, food availability and mortality are fundamental drivers of population dynamics and, therefore, key to understanding species coexistence under changing conditions.

Here, I study how environmental change alters conditions for coexistence of an endemic species flock. I use a size‐structured consumer‐resource model where at birth, individuals are constrained to feed on a common resource. As they mature, individuals diversify into specialized forms, each adapted to feed on specific resources. I model environmental change as either a shift in resource supply or a change in species‐specific mortality, and I explore how these factors impact species coexistence and the potential for species collapse.

I show that a change in environmental conditions affecting one species can trigger a cascade effect, altering the size structure of the focal species and subsequently affecting resource availability for other species. Coexistence of all species under these altered ecological conditions is no longer possible. Importantly, once species are lost, they cannot re‐establish even if environmental conditions return to their original state, resulting in irreversible biodiversity loss. While regime shifts, where ecosystems abruptly transition into a new state after crossing a critical threshold, are well‐known consequences of environmental change (Scheffer et al. 2001), this study provides a more mechanistic understanding of how species richness can be lost. I show that changes in the size structure of a single species can disrupt coexistence, ultimately driving community collapse. These findings underscore the vulnerability of endemic species flocks to environmental change and highlight the potential for unexpected outcomes in the face of shifting ecological conditions, particularly in the context of climate change.

## 2 Model and Analysis

I use the food web approach for size‐structured populations proposed by Hartvig et al. (2011) and used in ten Brink and Seehausen (2022). This model describes the processes of food encounter, somatic growth, reproduction, and mortality at the individual level, based on body mass *m* and niche trait value *x*_*i*_ (the index *i* indicates the species in a multispecies context). Somatic growth, reproduction, and starvation mortality all depend on food consumption. Model parameters are made species independent through scaling with individual body mass *m* and body mass at maturation *m*_mat_. Parameter values, provided in Table S1, are derived from allometric scaling laws based on cross‐species analyses of fish communities (Hartvig et al. 2011). The variables and individual‐level model equations are summarized in Table S2.

### 2.1 Life‐History

In the early stages of an adaptive radiation, species primarily diverge in trophic morphology while other life‐history traits remain similar (Gavrilets and Losos 2009; Streelman and Danley 2003). I therefore make the assumption that all species in the model have similar life‐history characters, and only differ in their trophic morphology. Individuals of all species are born with a body mass *m*_b_ and initially feed on a shared resource with density *R*_s_. Upon reaching a body mass *m*_shift_, they undergo a discrete diet shift at which they get access to six well‐mixed resources, each with a density *R*_*j*_ (where *j* = 1,2,…,6). I find qualitatively similar results with fewer or more resources (Figure S1). Since these resources require specific morphological adaptations to be effectively used (see below), making it impossible for individuals to specialize on multiple resources, I refer to them as species‐specific resources. Although individuals can potentially feed on all these resources, they will only utilize a few that require similar morphological adaptations. The larger individuals get, the more energy they allocate to reproduction and producing offspring. I refer to small juveniles (*m < m*_shift_) that feed on the shared resource and large individuals ($m\geq m_{\text{shift}}$) that feed on species‐specific resources. With the default parameters provided in Table S2, large individuals include both large juveniles (*m < m*_mat_) and reproducing adults ($m\geq m_{\mathrm{mat}}$). Due to the gradual allocation function (Equation 7), there is no distinct size threshold separating juveniles from adults; large juveniles already allocate some energy to reproduction, while adults continue to allocate energy to growth.

### 2.2 Species Niche and Developmental Trade‐Off

I assume that each resource, including the shared resource, requires a different morphology for efficient exploitation. As a result, consumers face a trade‐off such that adapting to one resource reduces efficiency on others. This also implies that specialization on one of the species‐specific resources comes at the expense of specialization on the shared resource, leading to a developmental trade‐off between early and late foraging success.

To model these assumptions, I assume that each consumer species *i* has a niche trait value *x*_*i*_, determining its resource specialization. Resources are characterized by trait values $\theta_{j}$, and the closer *x*_*i*_ is to $\theta_{j}$, the more efficient the consumer feeds on that resource and the less efficient on others. The attack rate of species *i* on resource *R*_*j*_ increases allometrically with body mass *m* following

(1) $$a_{i,j}\left(m,x_{i},\theta_{j}\right)=A_{i,j}\left(x_{i},\theta_{j}\right)m^{q},$$

where parameter q is a positive exponent signifying that larger individuals search a larger volume per unit time. The trait‐dependent attack rate coefficient $A_{i,j}\left(x_{i},\theta_{j}\right)$ depends on the resource *j* and the niche trait *x*_*i*_, reaching a maximum *A*_max_ when *x*_*i*_ matches the optimal trait value $\theta_{j}$. This coefficient decreases in a Gaussian manner as *x*_*i*_ deviates from $\theta_{j}$ following

(2) $$A_{i,j}\left(x_{i},\theta_{j}\right)=A_{\max}\;\exp\left[-{\left(x_{i}-\theta_{j}\right)}^{2}/\left(2\tau_{j}^{2}\right)\right].$$

The value for $\theta_{j}$ sets the trait value *x*_*i*_ at which the attack rate on resource *j* is highest, while $\tau_{j}$ controls how quickly the attack rate decreases as *x*_*i*_ deviates from $\theta_{j}$. Together, these parameters determine how efficient consumers exploit resources on which they are not specialized (i.e., when *x*_*i*_ $\neq \theta_{j}$). The closer the values $\theta_{j}$ are to each other and the wider the feeding curves (high $\tau_{j}$ values), the higher the attack rate individuals have on resources on which they are not specialised.

The widths of and the distance between the feeding curves indicate how much the species‐specific resources differ from each other. In line with previous work (see ten Brink and Seehausen 2022) I assume that the feeding curves for all species‐specific resources (Equation 2) have a width of $\tau_{j}=\tau =1$ but differ in their $\theta_{j}$ values. Here, $\theta_{1}$ equals 1, and each subsequent θ value increases by 2.5, with $\theta_{s}$ set at 0 (see Figure S2 in the appendix for results in case of different distances between feeding curves). This implies that species specialized on $R_{1}$ are the most efficient at exploiting the shared resource, while species specialized on $R_{6}$ are the least efficient. The width of the feeding curve of the shared resource, $\tau_{s}$, determines the strength of this developmental trade‐off between early and late foraging success. I assume a default value of $\tau_{s}$= 20, ensuring that even species specialized on $R_{6}$ remain relatively efficient on the shared resource (Figure S3).

### 2.3 Food Intake, Growth, Reproduction, and Mortality

Food intake is described by a Holling type II functional response, which is size‐specific with maximum intake denoted by *hm* ^*n*^ and equals

(3) $$I_{i,s}\left(m,x_{i},R_{s}\right)=hm^{n}\frac{a_{i,s}\left(m,x_{i},\theta_{s}\right)R_{s}}{hm^{n}+a_{i,s}\left(m,x_{i},\theta_{s}\right)R_{s}}$$

for individuals feeding on the shared resource *R*_s_ (*m < m*_shift_). For individuals feeding on the species‐specific resource *R*_*j*_ food intake equals

(4) $$I_{i,j}\left(m,x_{i},R\right)=hm^{n}\frac{a_{i,j}\left(m,x_{i},\theta_{j}\right)R_{j}}{hm^{n}+\sum_{j}a_{i,j}\left(m,x_{i},\theta_{j}\right)R_{j}},$$

with **R** = ($R_{s},R_{1},\ldots R_{6}$) denoting the resource vector. Total resource intake of an individual of species *i* and size *m* is then given as

(5) $$I_{i}\left(m,x_{i},R\right)=\left\{\begin{array}{ll} hm^{n}\frac{a_{i,s}\left(m,x_{i},\theta_{s}\right)R_{s}}{hm^{n}+a_{i,s}\left(m,x_{i},\theta_{s}\right)R_{s}} & \text{if}\;m<m_{\text{shift}},\\ hm^{n}\frac{\sum_{j}a_{i,j}\left(m,x_{i},\theta_{j}\right)R_{j}}{hm^{n}+\sum_{j}a_{i,j}\left(m,x_{i},\theta_{j}\right)R_{j}} & \text{otherwise}. \end{array}\right.$$

Ingested food $I_{i}\left(m,x_{i},R\right)$ is assimilated with efficiency α. Assimilated energy is first used for maintenance costs, which allometrically increase with body mass as $km^{p}$. The net energy production rate of individuals is given by the difference between energy intake and maintenance costs and equals

(6) $$E_{i}\left(m,x_{i},R\right)=\alpha I_{i}\left(m,x_{i},R\right)-km^{p}$$

Individuals allocate a fraction $\psi_{i}\left(m\right)$ of this energy to reproduction, with the remainder used for growth. Energy allocation to reproduction increases with mass following

(7) $$\psi \left(m\right)={\left[1+{\left(\frac{m}{m_{\mathrm{mat}}}\right)}^{-u}\right]}^{-1}{\left(\frac{\mathrm{\eta m}}{m_{\mathrm{mat}}}\right)}^{1-n}$$

Growth and reproduction occur only if energy intake ${\mathrm{\alpha I}}_{i}\left(m,x_{i},R\right)$ exceeds maintenance costs $km^{p}$. Somatic growth therefore equals
(8)

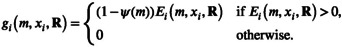

Reproduction rate equals
(9)

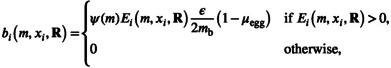

where parameter $m_{b}$ equals the size at birth, parameter ϵ the efficiency of offspring production, and the 2 in the denominator takes into account that only females spawn. Parameter $\mu_{\mathrm{egg}}$ is the fraction of eggs that die before hatching. The total birthrate of species *i* equals

(10) $$B_{i}\left(x_{i},R\right)=\int_{m_{b}}^{\infty }b_{i}\left(m,x_{i},R\right)N_{i}\left(m\right)\;dm$$

All individuals experience a size‐independent background mortality rate of $\mu_{b}$. If maintenance costs exceed energy intake, individuals die of starvation, with starvation mortality proportional to the energy deficiency and inversely proportional to fat reserves, which I assume to be a fraction ξ of the total body mass. Starvation mortality therefore equals
(11)

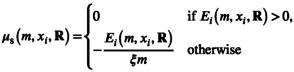

There is an additional size‐independent mortality $\mu_{\mathrm{add},i}$ for species *i* and a species and size‐specific mortality $\mu_{\text{size},i}$ for individuals with a body mass *m* between *m*_min_ and *m*_max_. Total mortality equals
(12)

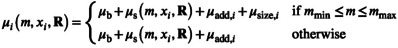




### 2.4 Resource and Consumer Dynamics

Resources follow semi‐chemostat dynamics (Persson et al. 1998) and reach densities of $R_{j,\max}$ in the absence of consumers. The productivity of a resource, denoted as ${\mathrm{\delta R}}_{j,\max}$, is the rate at which the resource is generated, in g per m^3^ per day. Unless otherwise stated, all species‐specific resources *R*_*j*_ have an equal productivity of ${\mathrm{\delta R}}_{c,\max}$ Consumers interact only indirectly with each other, via resource competition.

The dynamics of the shared resource *R*_s_, the species‐specific resources *R*_*j*_, and the population size distribution of species *i* are described by the following system of equations.

(13) $$\left\{\begin{array}{c} \begin{array}{c} \frac{dR_{s}}{dt}=\delta \left(R_{s,\max}-R_{s}\right)-\sum_{i}\int_{m_{b}}^{m_{\text{shift}}}I_{i,s}\left(m,x_{i},R_{s}\right)N_{i}\left(m\right)dm\\ \frac{dR_{j}}{dt}=\delta \left(R_{j,\max}-R_{j}\right)-\sum_{i}\int_{m_{\text{shift}}}^{\infty }I_{i,j}\left(m,x_{i},R\right)N_{i}\left(m\right)dm\\ \frac{\partial N_{i}\left(m\right)}{\partial t}+\frac{\partial g_{i}\left(m,x_{i},R\right)N_{i}\left(m\right)}{\partial m}=-\mu_{i}\left(m,x_{i},R\right)N_{i}\left(m\right) \end{array}\\ g_{i}\left(m_{b},x_{i},R_{s}\right)N_{i}\left(m_{b}\right)=B_{i}\left(x_{i},R\right). \end{array}\right.$$

### 2.5 Model Analysis

With six species‐specific resources available, six consumer species can potentially coexist in this system, each specialized on one of these resources. Since I want to study how environmental change can disrupt coexistence, I start with a community of six consumer species in an environment where such a species flock could have evolved. This implies that the resource supply of the shared resource is high and that the distances between the feeding curves are such that an adaptive radiation can unfold (ten Brink and Seehausen 2022). In Figure S2 of the appendix, I show results for conditions where a species flock could not have evolved but where coexistence is still possible, demonstrating that the results do not depend on the assumption that the community arose through evolutionary processes. To study the dynamics of the community over time, I use the Escalator Boxcar Train (EBT) method (de Roos 1988, 1997), a numerical approach for size‐structured population models.

To study how the community responds to environmental change, I first simulate a 10‐year period with stable environmental conditions, during which the community is in an equilibrium state. This is followed by a 40‐year period where the parameter of interest gradually changes with yearly steps. For visualisation purposes, the step size of the focal parameter is chosen such that the collapse happens after approximately 40 years; changing the step size does not qualitatively change the results. After these 40 years, the focal parameter is reset to its original value to study if the system can recover to its original state. For this aim, I implemented a small yearly invasion of newborn individuals (*m = m*_b_) for species that went extinct. Gradually reversing the focal parameter to its original value does not change results qualitatively.

To study the effect of changes in resource supply, I increase food productivity of resource 1 and 4 by adjusting parameters $R_{1,\max}$ and $R_{4,\max}$, respectively. The impact of changes in mortality are studied by increasing mortality for species 1, either for all individuals (parameter $\mu_{\mathrm{add},1}$) or specifically for small (parameter $\mu_{\text{size},1}$ with $m_{\min}=0$ and $m_{\max}=5$) or large (parameter $\mu_{\text{size},1}$ with $m_{\min}=5$ and $m_{\max}=100$) individuals. The appendix (Figure S4) includes an analysis of increased mortality for small individuals of all species (parameter $\mu_{\text{size},i}$ with $m_{\min}=0$ and $m_{\max}=5$).

In addition to the timeseries, I use the software package PSPManalysis (de Roos 2016, 2021b) to continue equilibria and detect bifurcation points where species go extinct. This software computes these bifurcation points as a function of two model parameters, which allows me to calculate the sensitivity of the results over large parameter ranges. Parameters studied are size at ontogenetic diet shift (parameter $m_{\text{shift}}$), productivity of the shared resource (parameter $R_{s,\max}$), strength of the developmental trade‐off (parameter $\tau_{s}$), and egg mortality ($\mu_{\mathrm{egg}}$). The underlying ecological model is for both approaches the same.

## 3 Results

### 3.1 Improving Conditions for a Focal Species Result in a Catastrophic Collapse of the Species Flock

**FIGURE 1:**
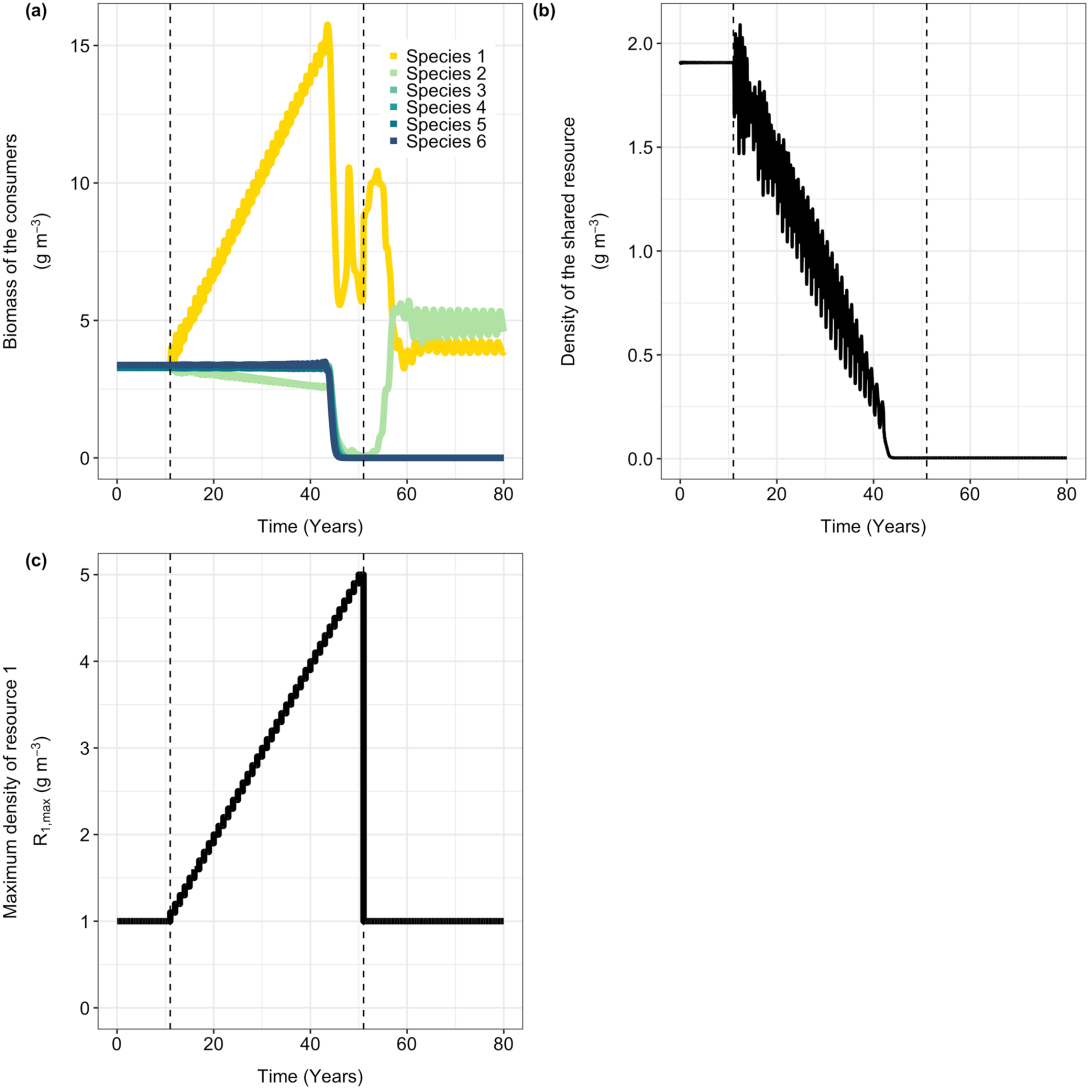
Timeseries of the densities (in g m^−3^) of the six species (a) and the shared resource (b) over time in a scenario where the productivity of resource 1 changes (c). For the first 10 years, the productivity of this focal resource is equal to the other 5 resources at $\delta R_{1,\max}$ = 0.1 g m^−3^ day^−1^. Over the next 40 years, the productivity gradually increases each year with steps of 0.01, until reaching a rate of 0.5 g m^−3^ day^−1^, after which it is restored to the original value of 0.1 g m^−3^ day^−1^. The vertical dashed lines indicate where the focal parameter starts to increase and when it is set back to its original value. Other parameters have default values.

I first study the response of the species flock to increases in the supply rate of the species‐specific resource on which species 1 is specialized ($\delta R_{1,\max}$). I vary productivity by changing parameter $R_{1,\max}$, while leaving the turnover rate δ unchanged. During the first 10 years, the supply rates of all six species‐specific resources are kept equal ($\delta R_{j,\max}=\delta R_{c,\max}$), resulting in a stable equilibrium with all six consumer species present (Figure 1).

As the productivity of the focal resource *R*_1_ increases over the following 40 years, the biomass of species 1 initially increases (Figure 1). Because large individuals of this species have more food available, they can grow faster and reproduce more, resulting in more newborn individuals and a larger population size. Although species 1 is specialized in feeding on resource 1, it also feeds slightly on resource 2 (*R*_2_). The higher biomass of species 1 increases competition for resource 2, which explains the slight decrease in the biomass of species 2. The other four species are not affected by the changes in the productivity of the focal resource, and their biomasses remain constant (Figure 1).

After approximately 45 years, there is a sudden shift in the community (Figure 1a), with all species except one rapidly declining and eventually going extinct. Restoring the supply rate of the focal species‐specific resource to its original level (Figure 1b) allows only species 2 to recover.

### 3.2 A Change in Size Distribution Leads to Strong Suppression of the Shared Resource, Preventing Re‐Establishment of Other Species

**FIGURE 2:**
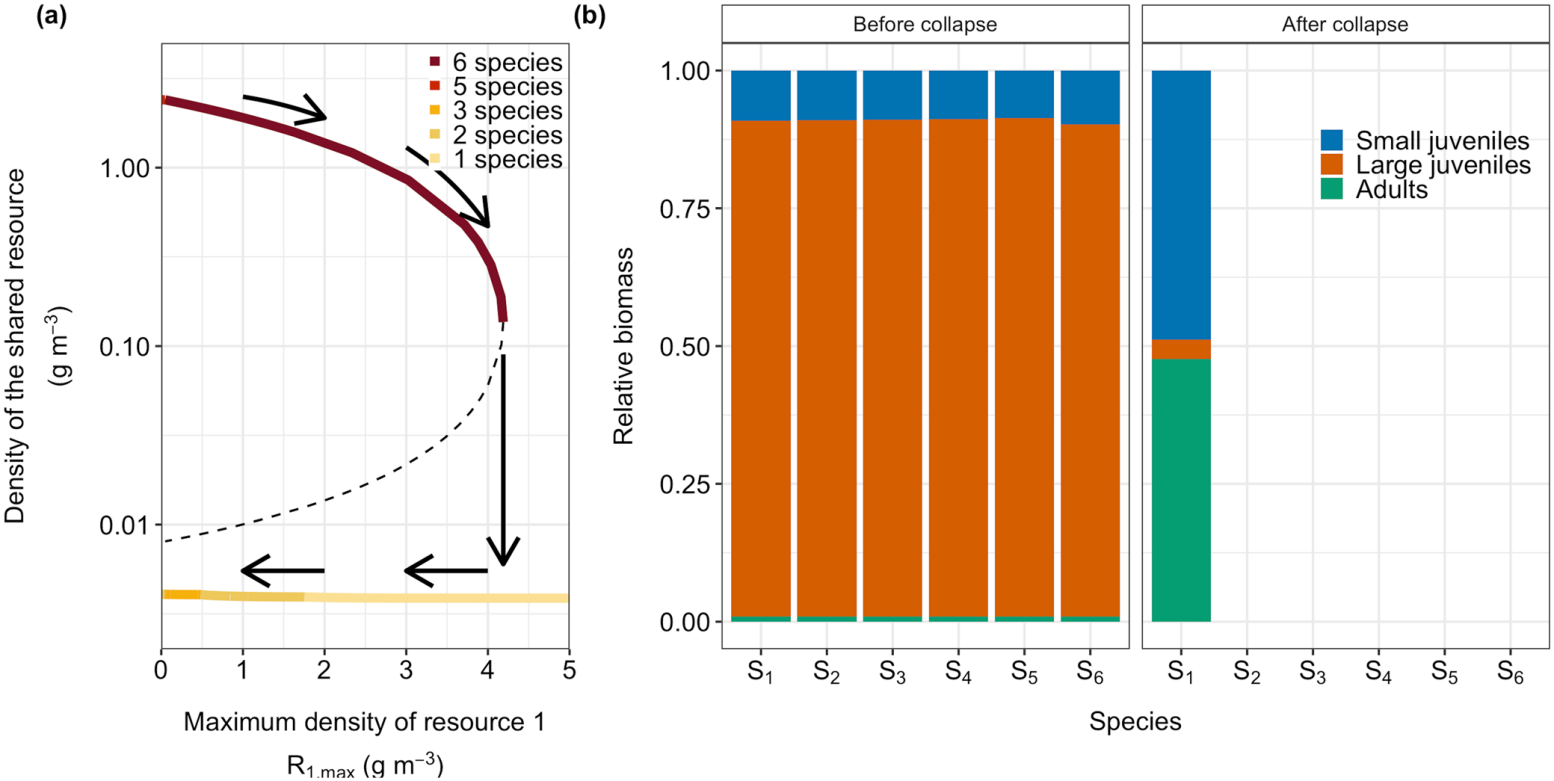
Panel (a) shows the density of the shared resource *R*_*s*_ (in g m^−3^), as a function of the maximum density of resource 1. Colors indicate the number of species that can coexist in the system. The solid lines indicate stable ecological equilibria, the dashed line indicates the unstable equilibrium. Arrows illustrate how increasing productivity shifts the system from six to one species and how the system remains in this state upon reversing productivity, demonstrating hysteresis. Panel (b) shows for each species the relative density of the three different size classes (small juveniles with *m* < *m*_shift_, large juveniles with $m_{\text{shift}}<m\leq m_{\mathrm{mat}}$, and adults with *m* ≥ *m*_mat_) just before and just after the collapse ($\delta R_{1,\max}$ = 0.42 g m^−3^ day^−1^ in both panels). Other parameters have default values.

To explain the catastrophic collapse, I show in Figure 2a the equilibrium density of the shared resource as a function of the supply rate of the focal species‐specific resource *R*_1_. Initially, with low supply rates of the focal resource, the density of the shared resource is high (Figure 2a). The size distribution of consumer species is dominated by large juveniles feeding on their species‐specific resource, and a relatively low biomass of small juveniles (Figure 2b). Due to the high density of the shared resource, small individuals have plenty of food available and grow quickly, gaining access to the species‐specific resources early in life.

As food availability for large individuals of the focal species increases, this species reproduces more, resulting in an increase in the number of offspring. This increases competition for the shared resource, which gradually declines with increasing productivity of the focal resource (Figure 2a). At a high value of $R_{1,\max}$, the composition of the focal species suddenly changes from being dominated by large juveniles to being dominated by small juveniles (Figure 2b), accompanied by a change from a high to a low density of the shared resource (Figure 2a). In this state, competition for the shared resource is intense, leading to the extinction of the other five species. When the productivity of the focal species‐specific resource is restored to its original level, the density of the shared resource remains low. Due to the developmental trade‐off some species (species 3, 4, 5, and 6) are more affected by early life competition than others (species 1 and 2), preventing their re‐establishment.

The sudden and irreversible biodiversity collapse is caused by ecological bistability. The system can either be in an equilibrium with all six species present and a high density of the shared resource, or in a state with fewer species, a low density of the shared resource, and consumer populations dominated by small juveniles. Note that for low values of $R_{1,\max}$ species 1 goes extinct in both equilibria (Figure 2a).

### 3.3 Even a Species With Increased Resource Availability Can Go Extinct

**FIGURE 3:**
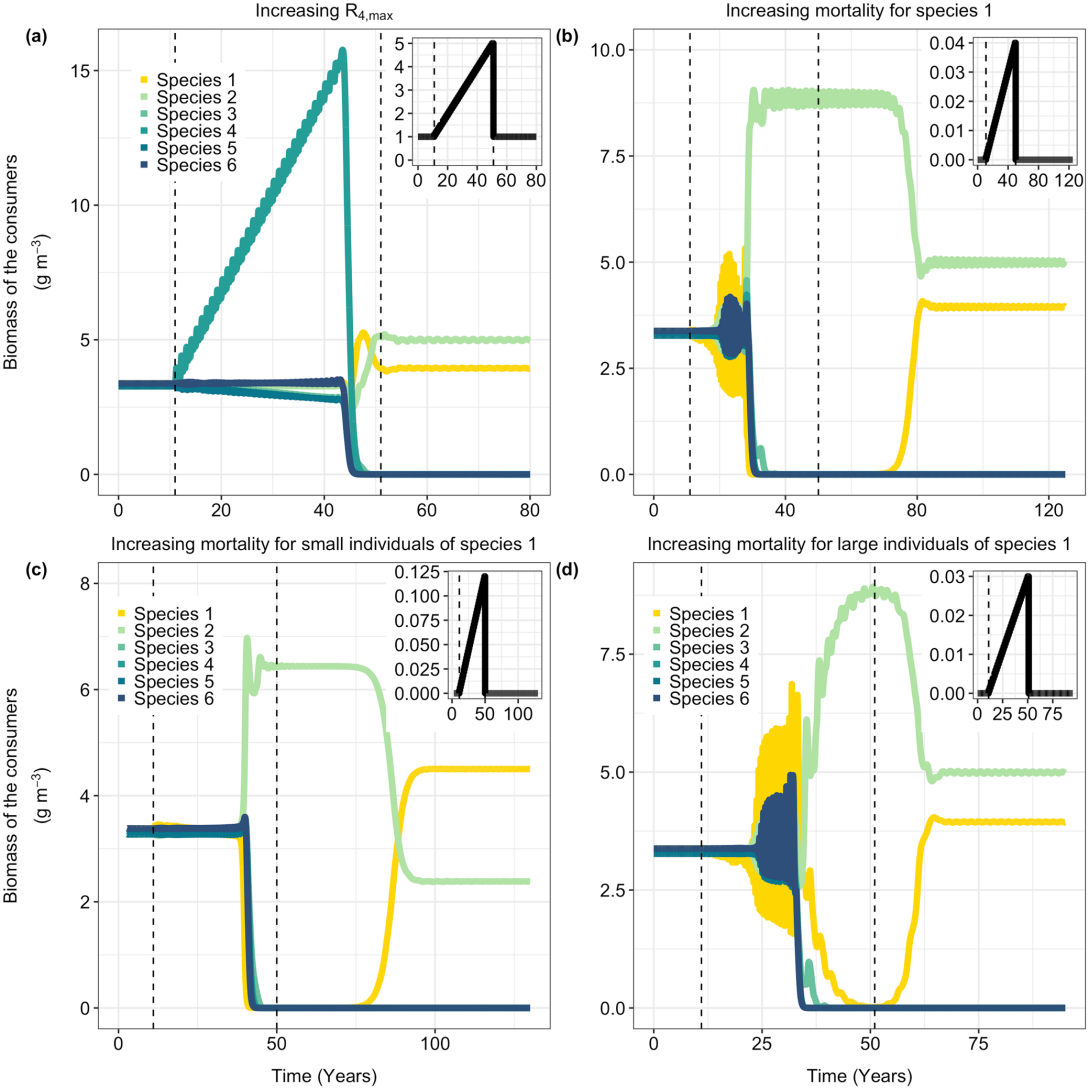
In panel (a) the productivity of species‐specific resource 4 is increased (${\mathrm{\delta R}}_{4,\max}$). In panels (b, c, and d), mortality for species 1 is increased. In panel (b) all size‐classes are affected ($\mu_{\mathrm{add},1}$), in panel c small individuals (*m < m*_shift_), and in panel d large individuals (*m* ≥ *m*_shift_). The insets in each panel show the change in the focal parameter, the vertical dashed lines indicate where the focal parameter starts to increase and when it is set back to its original value. In panel c, the size‐specific mortality affects small individuals of species 1 feeding on the shared resource ($\mu_{\min}=m_{b},\mu_{\max}=m_{\text{shift}}$). In this panel, the productivity of the shared resource is set at a value of 0.4 g m^−3^ day^−1^ since for higher values the system does not collapse for high mortality rates of small individuals of species 1 (Figure S5). In panel d, the size‐specific mortality affects large individuals feeding on the species‐specific resource ($\mu_{\min}=m_{\text{shift}},\mu_{\max}=100$). Other parameters have default values.

In the previous section, I showed that increasing food availability for species 1 led to the extinction of all other species. Here, I demonstrate that increasing food for a focal species, can, paradoxically, drive that particular species to extinction. In Figure 3a, I increase the productivity of the species‐specific resource for species 4 by increasing $R_{4,\max}$. The higher food availability for species 4 results in an increase in its biomass and a subsequent slight decrease in species 3 and species 5 due to increased competition for their species‐specific resources. As before, for high values of the focal species‐specific resource productivity (${\mathrm{\delta R}}_{4,\max}$), the system suddenly changes, leading to the extinction of most species, including species 4. The higher offspring production of species 4 increases competition for the shared resource, resulting in the collapse to the alternative equilibrium with low densities of the shared resource. Although species 4 has the most resources available when large, it is not the best competitor for the shared resource due to the developmental trade‐off and it is now outcompeted by species 1. When the productivity of the focal resource (${\mathrm{\delta R}}_{4,\max}$) is set back to its original value, species 2 can reinvade, but not the other four species (Figure 3a).

### 3.4 Increased Mortality Rates Result in a Catastrophic Collapse

In Figure 3, I show that increasing size‐independent mortality of species 1 ($\mu_{\mathrm{add},1}$, Figure 3b), or size‐specific mortality ($\mu_{\text{size},1}$) of either small ($m<m_{\text{shift}}$, Figure 3c) or large ($m\geq m_{\text{shift}}$, Figure 3c) individuals of species 1 also results in catastrophic collapses. Results are qualitively the same when mortality affects other species or multiple species (Figure S4).

When mortality is increased for all size classes of species 1 (Figure 3b), competition for the species‐specific resource *R*_*1*_ is reduced. This reduction in competition results in a higher population birth rate for this species. Although the total biomass of species 1 decreases slightly with higher mortality rates (Figure 3b), the increased birth rate leads to an increase in the biomass of newborns. This intensifies competition for the shared resource and reduces food availability for smaller individuals of all species, eventually leading to the collapse of the species flock into the alternative equilibrium (Figure 3b). Similarly, an increase in the mortality of large individuals also leads to higher population birth rates of species 1, eventually driving the system towards the alternative state where most species are extinct (Figure 3c). When mortality for species 1 is set back to the original level, this species will ultimately re‐invade the population (Figure 3b,c). The other species, however, are not able to recover due to the change in resource availability.

Increased mortality among small individuals of species 1 leads to a similar outcome (Figure 3d). As fewer small individuals survive to grow large enough to exploit the species‐specific resource, competition for food in the larger size classes of species 1 decreases, increasing the population birth rate of this species. This higher birth rate, in turn, results in more offspring and, ultimately, the collapse of the species flock.

### 3.5 Parameter Sensitivity

**FIGURE 4:**
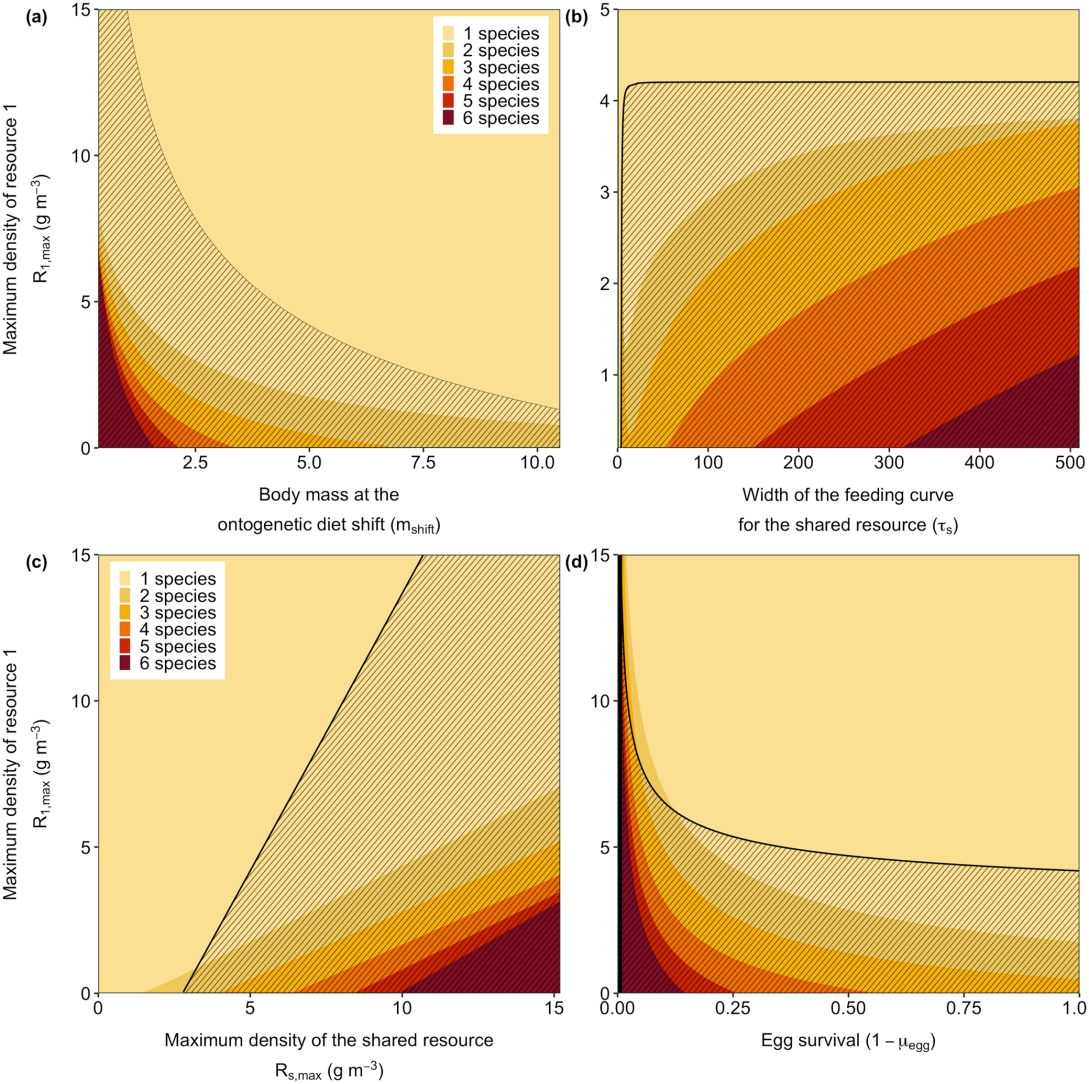
Two parameter plots showing the number of species that can coexist as a function of the maximum density of the focal resource ($R_{1,\max}$) and (a) the body mass at the ontogenetic diet shift (*m*_shift_), (b) the width of the feeding curve for the shared resource ($\tau_{s}$), (c) the maximum density of the shared resource ($R_{s,\max}$), and (d) the survival probability of eggs (1— $\mu_{\mathrm{egg}}$). Colors indicate the number of species that can coexist in the system. The hatched areas indicate the parameter areas where there is bistability between an equilibrium with six species or the alternative equilibrium with potentially less species (number of species indicated by the colors). Note that there are two equilibria with six species present (e.g., for high productivity of the shared resource and low productivity of the focal resource), where there is bistability in the size‐structure of the consumers but not in the number of species coexisting. Black areas indicate parameter values where none of the species can sustain a viable population. The size at the ontogenetic diet shift, *m*_shift_, equals 5 g in panel b, c, and d. The width of the feeding curve, $\tau_{s}$, equals 20 in panel a, c, and d. The productivity of the shared resource δ *R*_s,max_, equals 0.5 g m^−3^ day^−1^ in panels a, b, and d. The egg survival $\mu_{\mathrm{egg}}$ equals 1 in panels a, b, and c. Other parameters have default values.

The likelihood of a species collapse is sensitive to the body mass at the ontogenetic diet shift (Figure 4a), the strength of the developmental trade‐off (Figure 4b), the productivity of the shared resource (Figure 4c), and egg survival (Figure 4d). I show the sensitifity of these parameters as a function of the maximum resource density of *R*_1_ ($R_{1,\max}$), results are qualitatively similar under increased mortality rates.

When individuals shift to the species‐specific resources at a smaller body mass, their dependence on the shared resource is reduced. Therefore, competition for this resource becomes less strong, which makes competitive exclusion early in life less likely. Therefore, early diet shifts make the system more resilient to changes in resource productivity (Figure 4a).

While the width of the feeding curve for the shared resource does hardly influence the productivity level of the focal resource at which the collapse occurs (Figure 4b), it affects the number of species coexisting in the alternative equilibrium. A wider feeding curve (high value of $\tau_{s}$) corresponds to a weaker developmental trade‐off (Figure S3). A weak trade‐off means that different species are almost similar when small, leading to near‐neutral competition for the shared resource and allowing for greater species coexistence. Conversely, with narrow feeding curves (low $\tau_{s}$), bistability disappears, and only species 1 can persist in the system. In this extreme case, the trade‐off is so strong that individuals specializing in resources other than *R*_1_ have such a low attack rate on the shared resource that they are unable to reach adulthood.

High productivity of the shared resource makes the system more resilient to changes in productivity levels of the species‐specific resources (Figure 4c). For low productivity of the shared resource, the bistability disappears and only 1 or 2 species can coexist. As with a strong trade‐off (Figure 4b), with low productivity of the shared resource, there is not enough food available for some species to reach adulthood.

**FIGURE 5:**
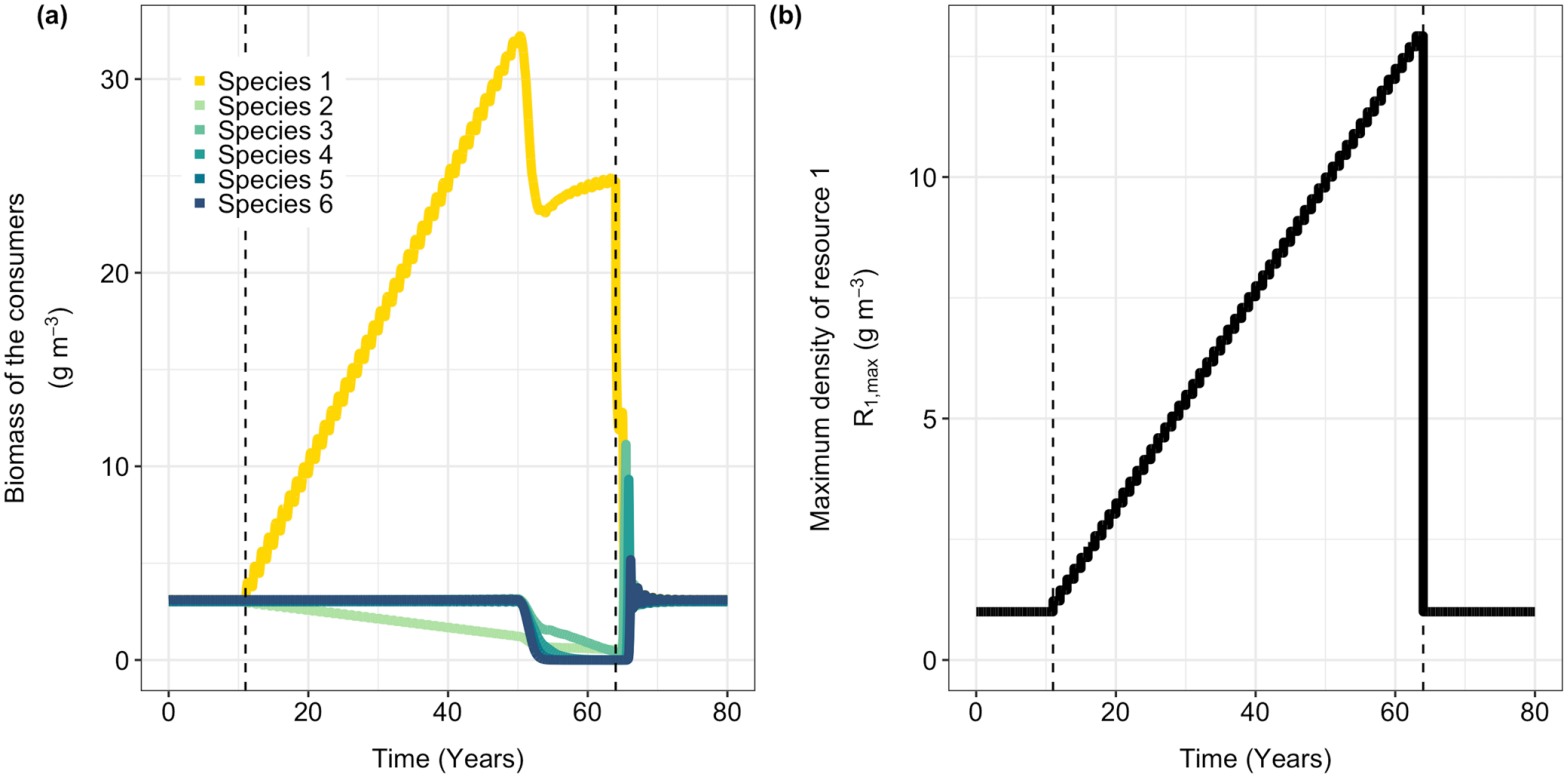
Timeseries of the densities (in g m^−3^) of the six species over time (a) in a scenario where the productivity of resource 1 increases (b). For the first 10 years, the productivity of this focal resource is equal to the other 5 resources at $\delta R_{1,\max}$ = 0.1 g m^−3^ day^−1^. Over the next 54 years, the productivity gradually increases each year with steps of 0.225, until reaching a rate of 1.3 g m^−3^ day^−1^, after which it is restored to the original value of 0.1 g m^−3^ day^−1^. The vertical dashed lines indicate where the focal parameter starts to increase and when it is set back to its original value. Egg survival, 1— $\mu_{\mathrm{egg}}$, equals 0.025, other parameters have default values.

Lower egg survival makes the system less prone to an irreversible collapse (Figure 4d). With high egg mortality, fewer individuals will hatch, resulting in relatively little competition for the shared resource. While increased productivity rates of the species‐specific resource will increase the birth rate of species 1 and thereby intensify competition, the collapse will only happen when the productivity of the focal resource is very high. While many species will go extinct when the productivity of the focal resource is high, for low egg survival, this collapse is not irreversible. When the productivity of the focal resource is set back to its original, low value, species are able to reinvade (Figure 5).

## 4 Discussion

A key consequence of environmental change is the potential for ecosystems to shift abruptly into a new state when environmental conditions cross a critical threshold (Scheffer et al. 2001). This study shows that an adaptive radiation can undergo such a regime shift and suddenly and irreversibly lose much of its biodiversity due to changes in the size structure of a single species within the flock. Increases in mortality or food availability for a focal species can shift the population from being dominated by large individuals to being dominated by small individuals, a common feature in models with ontogenetic diet shifts (de Roos and Persson 2013; Guill 2009; Schreiber and Rudolf 2008). This shift in the size structure of the focal species increases competition for the resource that newborn individuals of all species feed on, resulting in the extinction of multiple species due to competitive exclusion early in life. Species flocks in which individuals spend a long period on the shared resource, or that have strong development trade‐offs between the juvenile and adult morphology, are especially vulnerable. On the other hand, in systems with abundant food for small individuals or with low egg survival, species flocks are more resilient to environmental change and less likely to collapse.

Although there are several known cases of sudden species loss in adaptive radiations, including Hawaiian honeycreepers (Paxton et al. 2016), cichlids in Lake Victoria (Witte et al. 1992), and lemurs in Madagascar (Crowley et al. 2012), pinpointing the exact causes of these collapses is challenging. Studies on biodiversity loss often have to rely on fossils (e.g., Ngoepe et al. 2024) or rare observations (e.g., Ismail et al. 2014) to reconstruct the past and assess the current status of species flocks. While it is therefore difficult to find direct evidence for the mechanism presented here, the model provides insights on life‐history characteristics that make some species more likely to collapse.

The results presented here rely on the assumption that multiple coexisting species share a diet early in life before they switch to species‐specific resources. While this assumption is likely valid for many fish species (Chouinard and Bernatchez 1998; Damerau et al. 2012; Nunn et al. 2012; Sánchez‐Hernández et al. 2019; van Zwieten et al. 2016; Wellenreuther and Clements 2008; Wood et al. 1999), it is not applicable to all taxa. In many Lepidoptera species, for example, larvae are highly specialized in feeding on different resources (Altermatt and Pearse 2011; Braby and Trueman 2006), while adults primarily focus on dispersal and reproduction. In such cases, the assumption of a shared resource early in life does not apply. Additionally, although amphibians like frogs have tadpoles that are often generalist suspension feeders and probably share similar resources (but see Altig et al. 2007), individuals can disperse during the terrestrial phase, leading to potential spatial separation between species. This reduces the likelihood of a collapse as described here, especially since populations are seldom limited to a single pond, so if a collapse occurs, it will likely be local and not directly result in global extinction.

One major caveat in empirical studies of resource and habitat partitioning among ecotypes is the lack of consideration of ontogenetic niche shifts, despite the fact that most animal species change niche during ontogeny (Werner and Gilliam 1984). For instance, while anole adaptive radiations are well‐documented in the Greater Antilles, little is known about whether ecomorphs differ in prey type and size, and how their diet and habitat change during ontogeny (Losos 2009). Given the importance of ontogenetic diet shifts for diversification (Saltini et al. 2023; ten Brink and Seehausen 2022) and persistence of adaptive radiations, it is crucial that empirical studies of ecotypes investigate changes in diet and habitat throughout an organism's life.

An example that closely reflects the predicted collapse in the model is the decline of the *Labeobarbus* species flock in Lake Tana, Ethiopia. This species flock is the result of an adaptive radiation, where each species exhibits strong morphological adaptations to exploit different food resources (Sibbing and Nagelkerke 2001). As juveniles, fish species primarily consume insect larvae and zooplankton (Nagelkerke and Sibbing 2000), while as adults, each species specializes in distinct resources. The rapidly intensifying pressures on the Lake Tana ecosystem, particularly from fishing, have led to a sudden decline in both the abundance and diversity of *Labeobarbus* species, with some species now rarely caught (De Graaf et al. 2004; Gebremedhin et al. 2019). Although all size classes have been affected, the sharp decline in the number of large juveniles (De Graaf et al. 2004) is particularly notable, aligning with the model results presented here.

In summary, species flocks that share resources and habitats early in life and have limited dispersal potential are particularly vulnerable to catastrophic collapses triggered by a shift in the size structure of a single species. This makes endemic freshwater fish species particularly prone to such collapses. Moreover, freshwater ecosystems are among the most threatened on Earth (Albert et al. 2021) and are undergoing rapid changes due to climate change and other anthropogenic pressures that could trigger the type of collapse outlined in this study.

Climate change may alter food availability for fish species in freshwater lakes through both direct and indirect mechanisms, potentially impacting endemic species flocks. Direct effects are, for example, shifts in zooplankton communities as rising temperatures favor thermally tolerant species (Carter et al. 2017), and range shifts, with cold‐water organisms being generally negatively affected and warm‐water organisms positively affected (Heino et al. 2009). Indirect effects include increased terrestrial dissolved organic matter, resulting in lake ‘browning’ (Solomon et al. 2015). Browning reduces light and increases nutrient supply, which decreases benthic and increases pelagic primary production, impacting benthic invertebrates and other organisms that contribute to the food base (Hamdan et al. 2021; Vasconcelos et al. 2019). Such complex interactions will reshape relative availability and quality of food in lake ecosystems. Some changes, such as higher productivity of food sources like zooplankton, may increase the system's resilience by providing more resources for smaller individuals. In contrast, shifts in the relative food availability for large individuals could lead to the collapse of the species flock.

Climate change will alter mortality regimes in freshwater ecosystems as rising water temperatures increase ectotherm mortality due to higher metabolic demands (Munch and Salinas 2009). Additionally, shifting climatic conditions may facilitate invasions by exotic species (Walther et al. 2009), which can intensify predation on native fish. However, my model suggests that increased mortality alone does not necessarily lead to catastrophic collapses. For example, predation by the invasive round goby ( *Neogobius melanostomus* ) on the eggs of native fish (Kornis et al. 2012) could reduce early‐life competition and make species flocks more resilient. Predicting how climate change will impact endemic species flocks remains challenging and requires extensive knowledge on the ecology of such species.

In this study, I focused on the ecological effects of environmental change, excluding evolutionary processes. Evolutionary change can influence the likelihood of tipping points (Chaparro‐Pedraza 2021; Chaparro‐Pedraza and de Roos 2020) and may lead to diversification processes that could increase biodiversity after a collapse (Frei et al. 2023; Hirsch et al. 2013). However, it is unlikely that in the current model evolutionary changes in specialization traits would alter the tipping conditions or lead to diversification after the collapse. Before the collapse, all species are specialized on their species‐specific resource and there is during environmental change little selection pressure to modify these traits. After the collapse, the abiotic conditions change in such a way that disruptive selection among large individuals is insufficient to drive diversification (ten Brink and Seehausen 2022).

The catastrophic collapse described here results from competitive exclusion during early life stages, which arises from the developmental trade‐offs in feeding efficiency. Species with ontogenetic diet shifts face a fundamental trade‐off between early and later life performance, as different food types often require different morphologies (Andersson 2003; Ebenman 1992; Hjelm et al. 2000; Svanbäck and Eklöv 2003; Werner 1977). One possibility is that species evolve traits to minimize this developmental trade‐off and thereby avoid competitive exclusion, for example by producing larger eggs. However, before the collapse, small individuals experience little competition, which means there is minimal selection pressure to improve feeding efficiency during this life phase. After the sudden change in abiotic conditions, selection to improve early‐life feeding efficiency is strong. However, evolutionary rescue is only possible when species adapt quickly enough, which requires sufficient genetic diversity. Future research could investigate the potential and conditions for such evolutionary rescue.

The assumption that species share resources early in life and specialize later also applies to other communities that do not necessarily form a species flock, such as fish species coexisting in the same lake. However, an important model assumption here is that all species have highly similar life histories, including identical size at birth and maturation. This additional assumption makes the findings presented here most applicable to young adaptive radiations where species have mainly diverged in trophic morphology (Gavrilets and Losos 2009; Streelman and Danley 2003). Whether the results extend to systems where non‐related species share the same resource early in life but differ in other life history traits such as size at birth could be explored in future research.

In conclusion, my results show that endemic species flocks are highly sensitive to extinction, where environmental change affecting a single species can trigger a cascade, altering the size structure of the focal species and subsequently affecting resource availability for other species. Under these altered conditions, coexistence of all species becomes impossible, resulting in irreversible biodiversity loss. This study adds to the growing body of research emphasizing the critical role of intraspecific diversity in ecosystems, as it shapes key processes such as coexistence (Anaya‐Rojas et al. 2021; de Roos 2021a), competition (Moccetti et al. 2024; Uszko et al. 2022), and diversification (Butler et al. 2007; Chaparro‐Pedraza 2024; Saltini et al. 2023; ten Brink and Seehausen 2022). With rapid environmental changes driven by climate change and other anthropogenic factors, understanding how intraspecific diversity contributes to species persistence will be essential for conserving biodiversity in the future.

## Author Contributions

**Hanna ten Brink:** conceptualization, formal analysis, investigation, methodology, validation, visualization, writing – original draft, writing – review and editing.

## Conflicts of Interest

The author declares no conflicts of interest.

## Supporting information


Data S1.

## Data Availability

The code and scripts that support the findings of this study are openly available in Zenodo at https://doi.org/10.5281/zenodo.15283235.
