# Supplementary material for: Environmental Change Can Result in Irreversible Biodiversity Loss in Recently Formed Species Flocks
Source: Glob Chang Biol. 2025 May 10;31(5):e70239. doi: 10.1111/gcb.70239 (PMC12065021; doi:10.1111/gcb.70239)
Supplement: Supplementary file 1 — Data S1. [file GCB-31-e70239-s001.pdf]

Supporting information

Table S1: Model variables, parameters, and their default value from Hartvig *et al.* (2011)

Variable	Description	Value	Unit
	**Variables**		
$N_i(m)$	Consumer size distribution of species i	-	
m	Size of a consumer	-	g
R_s	Density of the shared resource	-	g m^{-3}
R_j	Density of resource j	-	g m^{-3}
	**Feeding**		
q	Exponent for search volume	0.8	-
n	Exponent for maximum food intake	0.75	-
h	Maximum food intake	0.233	g^{1-n} day^{-1}
A_max	Maximum size-specific attack rate	21.9	m^3 g^{-q} day^{-1}
τ_s	Width of the attack rate function for the shared resource	20	-
τ	Width of the attack rate function for species-specific resources	1	-
θ_s	Optimal trait value of R_s	0	-
θ_1	Optimal trait value of R_1	1	-
θ_2	Optimal trait value of R_2	3.5	-
θ_3	Optimal trait value of R_3	6	-
θ_4	Optimal trait value of R_4	8.5	-
θ_5	Optimal trait value of R_5	11	-
θ_6	Optimal trait value of R_6	13.5	-
	**Species niche**		
x_1	Trait of species 1	$\theta_1(= 1)$	-
x_2	Trait of species 2	$\theta_2(= 3.5)$	-
x_3	Trait of species 3	$\theta_3(= 6)$	-
x_4	Trait of species 4	$\theta_4(= 8.5)$	-
x_5	Trait of species 5	$\theta_5(= 11)$	-
x_6	Trait of species 6	$\theta_6(= 13.5)$	-
	**Growth and Reproduction**		
m_b	Body mass at birth	0.5	mg
m_shift	Body mass at ontogenetic shift	5	g
m_mat	Body mass at maturation	25	g
η	Body mass at maturation relative to asymptotic mass	0.25	-
u	Width of maturation transition	10	-
ϵ	Efficiency of offspring production	0.1	-
α	Assimilation efficiency	0.6	-

k	Standard metabolism	0.0274	g^{1-p} day^{-1}
p	Exponent of standard metabolism	0.75	-
	**Mortality**		
ξ	Fraction of energy reserves	0.1	-
μ_{b}	Background mortality rate	0.01	day^{-1}
$\mu_{\mathrm{add},\,i}$	Additional, size-independent mortality for species *i*	0	day^{-1}
$\mu_{\mathrm{size},\,i}$	Size-specific mortality for species *i*	0	day^{-1}
μ_{egg}	Egg mortality	0	-
m_{min}	Minimum size threshold for size-specific mortality	0	g
m_{max}	Maximum size threshold for size-specific mortality	100	g
	**Resource dynamics**		
δ	Resource turnover rate	0.1	day^{-1}
$R_{\mathrm{s,max}}$	Maximum density of the shared resource	5	$\mathrm{g\ m}^{-3}$
$R_{j,\mathrm{max}} = R_{\mathrm{c,max}}$	Maximum density of each (non-focal) species-specific resource	1	$\mathrm{g\ m}^{-3}$

Table S2: Individual-level equations for both the deterministic and individual based model.

**Function**	**Equation**
Maximum resource-specific attack rate coefficient	$A_{i,j}(x_i,\theta_j) = A_{\mathrm{max}} \exp\left[-(x_i-\theta_j)^2/(2\tau_j^2)\right].$
Size- and resource-specific attack rate	$a_{i,j}(m,x_i,\theta_j) = A_{i,j}(x_i,\theta_j)m^q$
Maximum size-specific food intake	hm^n
Intake of the shared resource R_{s}	$I_{i,\mathrm{s}}(m,x_i,R_{\mathrm{s}}) = hm^n \dfrac{a_{i,\mathrm{s}}(m,x_i,\theta_{\mathrm{s}})R_{\mathrm{s}}}{hm^n + a_{i,\mathrm{s}}(m,x_i,\theta_{\mathrm{s}})R_{\mathrm{s}}}$
Intake of species-specific resource *Rj*	$I_{i,j}(m,x_i,\mathbf{R}) = hm^n \dfrac{a_{i,j}(m,x_i,\theta_j)R_j}{hm^n + \sum_j a_{i,j}(m,x_i,\theta_j)R_j}$
Total food intake (Holling type 2 functional response for multiple prey)	$I_i(m,x_i,\mathbf{R}) = \begin{cases} hm^n \dfrac{a_{i,\mathrm{s}}(m,x_i,\theta_{\mathrm{s}})R_{\mathrm{s}}}{hm^n + a_{i,\mathrm{s}}(m,x_i,\theta_{\mathrm{s}})R_{\mathrm{s}}} & \text{if } m < m_{\mathrm{shift}}, \\ hm^n \dfrac{\sum_j a_{i,j}(m,x_i,\theta_j)R_j}{hm^n + \sum_j a_{i,j}(m,x_i,\theta_j)R_j} & \text{otherwise} \end{cases}$
Maintenance costs	km^p
Net energy production	$E_i(m,x_i,\mathbf{R}) = \alpha I_i(m,x_i,\mathbf{R}) - km^p$

**Function**	**Equation**
Fraction of energy allocated to reproduction	$\psi(m) = \left[1 + \frac{m}{m_{\mathrm{mat}}}^{-u}\right]^{-1} \left(\frac{\eta m}{m_{\mathrm{mat}}}\right)^{1-n}$
Somatic growth	$g_i(m, x_i, \mathbf{R}) = \begin{cases} \big(1 - \psi(m)\big) E_i(m, x_i, \mathbf{R}) & \text{if } E_i(m, x_i, \mathbf{R}) > 0, \\ 0 & \text{otherwise} \end{cases}$
Reproduction rate	$b_i(m, x_i, \mathbf{R}) = \begin{cases} \psi(m) E_i(m, x_i, \mathbf{R}) \frac{\epsilon}{2 m_{\mathrm{b}}} (1 - \mu_{\mathrm{egg}}) & \text{if } E_i(m, x_i, \mathbf{R}) > 0, \\ 0 & \text{otherwise} \end{cases}$
Total offspring production per species	$B_i(x_i, \mathbf{R}) = \int_{m_{\mathrm{b}}}^{\infty} b_i\,(m, x_i, \mathbf{R}) N_i(m)\,\mathrm{d}m$
Starvation mortality rate	$\mu_{\mathrm{s}}(m, x_i, \mathbf{R}) = \begin{cases} 0 & \text{if } E_i(m, x_i, \mathbf{R}) > 0, \\ -\frac{E_i(m, x_i, \mathbf{R})}{\xi m} & \text{otherwise} \end{cases}$
Total mortality rate	$\mu_i(m, x_i, \mathbf{R}) = \begin{cases} \mu_{\mathrm{b}} + \mu_{\mathrm{s}}(m, x_i, \mathbf{R}) + \mu_{\mathrm{add},\, i} + \mu_{\mathrm{size},\, i} & \text{if } m_{\mathrm{min}} \leq m \leq m_{\mathrm{max}} \\ \mu_{\mathrm{b}} + \mu_{\mathrm{s}}(m, x_i, \mathbf{R}) + \mu_{\mathrm{add},\, i} & \text{otherwise} \end{cases}$

Different species-specific resources

In the main text, I chose six resources to ensure the community is large enough to study multispecies interactions while remaining computationally feasible. Here, I show that the results presented in the main text are qualitatively the same in case of a different number of species-specific resources available (Fig. S1). The number of species-specific resources available determines the maximum number of coexisting species in the system. Increasing the productivity of a focal species-specific resource will ultimately result in the collapse of the species flock (Fig. S1). The more resources available, the faster a collapse occurs. This is because when more species coexist, the higher competition for the shared resource is.

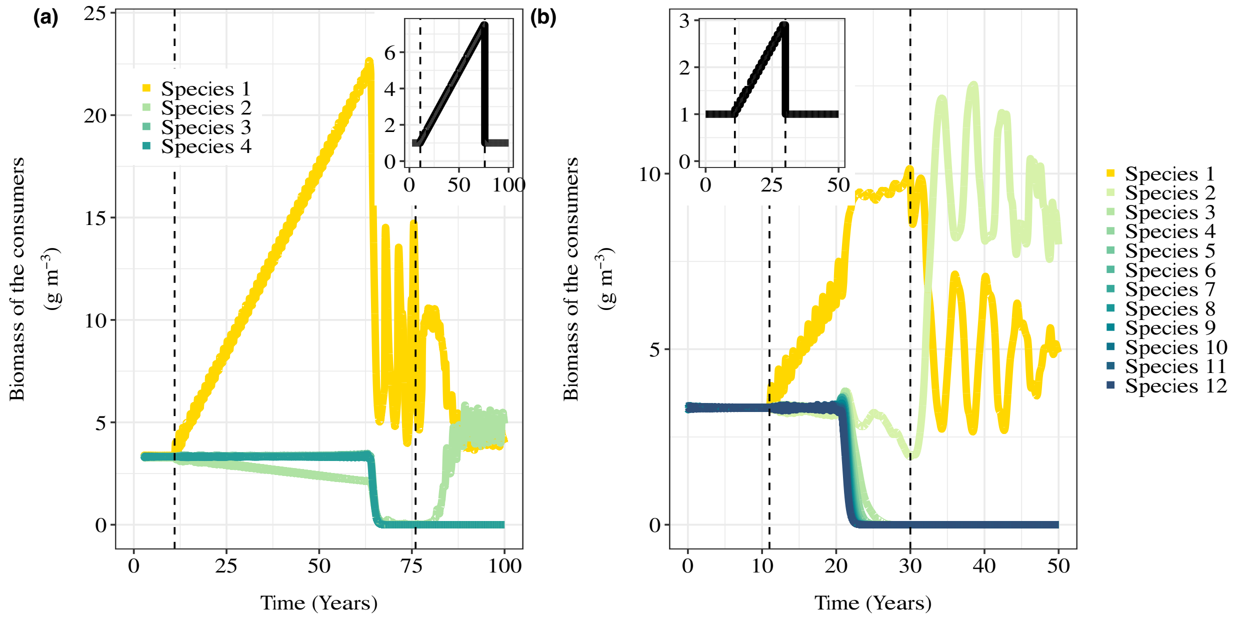

Figure S1: In panel a) there are in total four species-specific resources available, resulting in a maximum of four coexisting species. In panel b) there are twelve species-specific resources available, resulting in a maximum of twelve coexisting species. The insets in each panel show the change in the focal parameter, the vertical dashed lines indicate where the focal parameter starts to increase and when it is set back to its original value. The trait of species *i* equals $x_i = \theta_i = 1 + 2.5 * (i - 1)$. The productivity of the shared resource equals 0.5 g m^{-3} day^{-1} in panel a, and 0.7 g m^{-3} day^{-1} in panel b. Other parameters have default values.

Different distances between the feeding curves

The widths of the feeding curves and the distance between them indicate how much the species-specific resources differ from each other. Curves that are far away from each other imply that individuals require highly specific morphologies to efficiently utilize one of the resources. For an adaptive radiation to unfold with the chosen parameters, the distance between the curves should be between 1.9 and 4.2 (ten Brink and Seehausen 2022). I therefore chose a distance of 2.5 between the feeding curves of the species-specific resources in the main text. Here, I show results in case the curves are relatively close (distance of 1, Fig. S2a) or far (distance of 5, Fig. S2b) from each other. Since these distances would never result in an adaptive radiation, the community of coexisting species in these cases originated differently (e.g., via multiple immigration events).

In case of feeding curves close to each other, only four species can coexist in the system. The reason is that species specialized on a particular species-specific resource ($x_i = \theta_j$) can also feed efficiently on nearby resources. For example, a species specialized on resource 5 also feeds efficiently on resources 4 and 6. However, species specialized on the boundary resources (1 and 6) are at a disadvantage, as they have access to fewer alternative resources compared to species in the middle. As a result, these boundary species are outcompeted by species specialized on centrally located resources, leading to a community where only four species persist.

Figure S2 shows that the distance between the feeding curves does not qualitatively change the results. As before, changes in resource productivity of a focal resource can result in the collapse of the community. In case of close feeding curves (Fig. S2a), species 3 initially profits the most from the increase in the productivity of resource 1. However, this species goes extinct after the collapse to the alternative equilibrium, since it is not the best competitor for the shared resource.

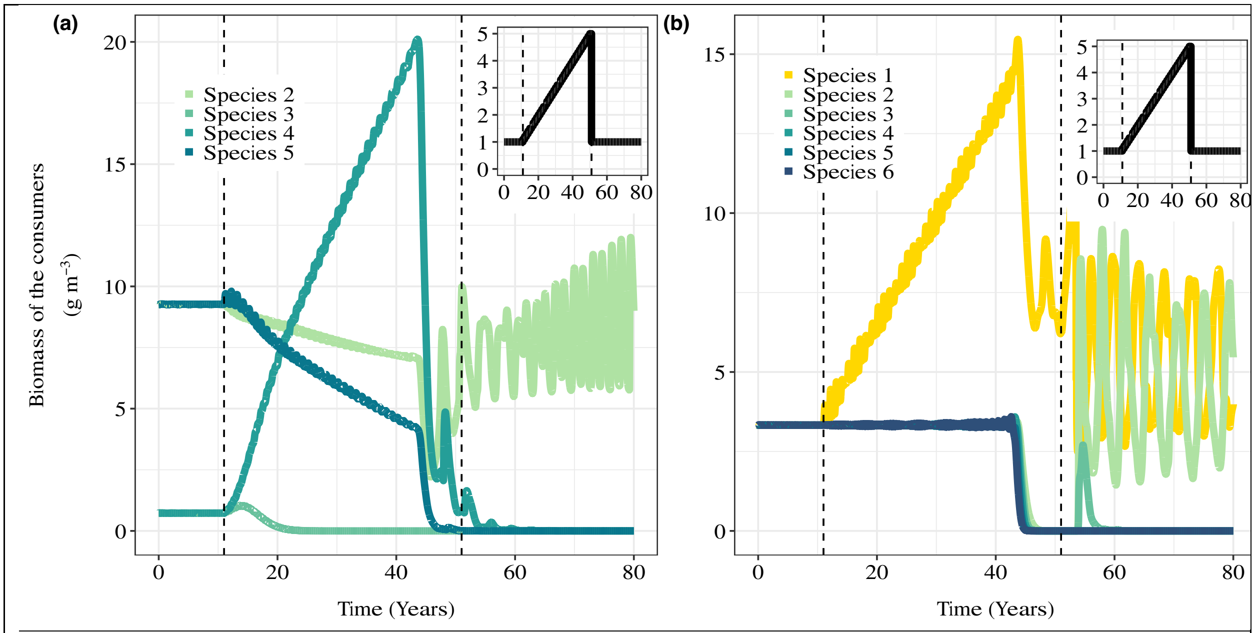

Figure S2: Timeseries of the densities (in g m^{-3}) of four (a) or six species (b) over time in a scenario where the productivity of resource 1 increases. For the first 10 years, the productivity of this focal resource is equal to the other 5 resources at $\delta R_{1,\max}$= 0.1 g m^{-3} day^{-1}. Over the next 50 years, the productivity gradually increases each year with steps of 0.01, until reaching a rate of 0.5 g m^{-3} day^{-1}, after which it is restored to the original value of 0.1 g m^{-3} day^{-1}. The insets in each panel show the change in the focal parameter, the vertical dashed lines indicate where the focal parameter starts to increase and when it is set back to its original value. The distance between the species-specific feeding curves equals 1 (panel a) and 5 (panel b). Other parameters have default values.

Feeding curves

Figure S3 shows how the attack rate coefficient $A_{i,j}$ (Eq. 2) changes as a function of the specialization trait x_i for each of the seven resources. In case of the default developmental trade-off (τ_s= 20), species specializing on R_1 suffer much less from this trade-off compared to species specializing on R_6 (Fig. S3a). For wider feeding curves (e.g., τ_s = 200, Fig. S3b), these differences are very small.

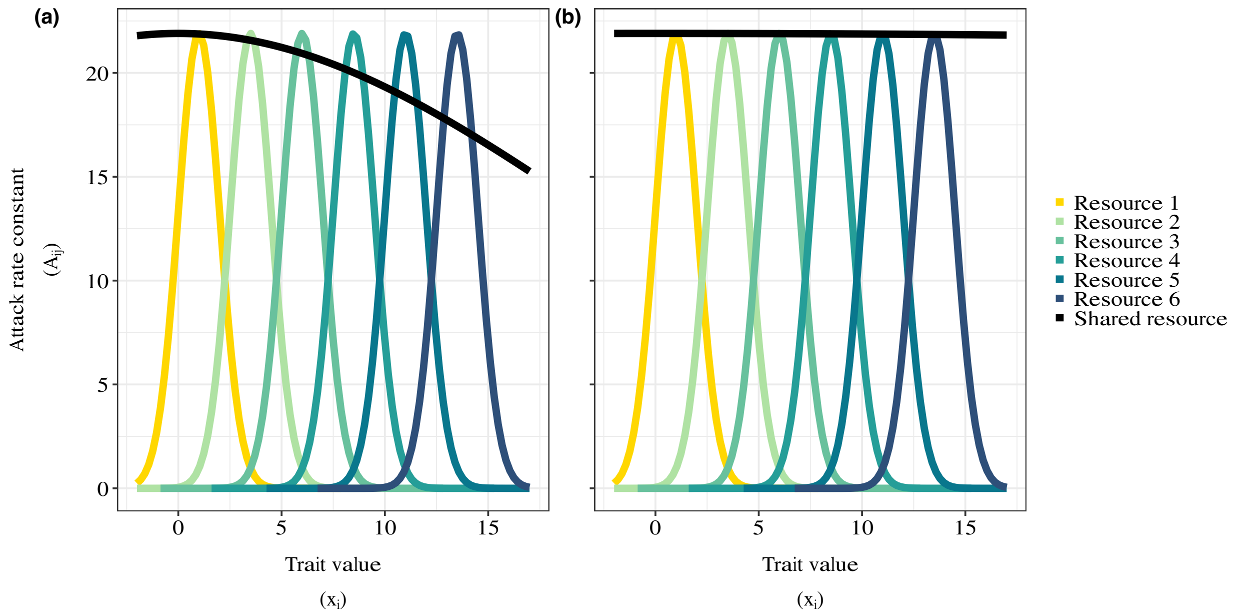

Figure S3: The attack rate constant, $A_{i,j}$, as a function of an individual's trait value, x_i, for all 7 resources (one shared resource (black) and six species-specific resources). In panel (a) τ_S= 20 (default), in panel (b) τ_S= 200. When τ_S= 20, the attack rate constant of an individual specialized on resource 1 (x_i=1) equals 21.9, and for an individual specialized on resource 6 (x_i=13.5) it equals 17.4. In case τ_S= 200, species 1 has an attack rate constant of 21.9 and species 6 one of 21.85. All other parameter values have default values.

Effects of mortality

Here, I show that increased productivity of the shared resource makes ecosystems more resilient to catastrophic collapses caused by mortality (Fig. S4 and S5). Furthermore, I demonstrate that the results remain robust when mortality affects all species rather than just one (Fig. S4).

When (size-specific) mortality affects all species, a sudden and irreversible collapse can occur (Fig. S4a). However, with higher resource productivity, the system becomes more resilient (Fig. S4b). In this scenario, a collapse only happens when mortality levels are very high, furthermore, many species can successfully recolonize the system after mortality levels are set back to the original value.

When a single species experiences elevated levels of (size-specific) mortality, it can result in a sudden and irreversible species collapse (Fig. 3 in main text, Fig. S5a). However, this outcome changes when the shared resource has high productivity levels (Fig. S5b). While size-specific mortality will still result in an increase of the number of small individuals, the high resource productivity prevents competitive exclusion by maintaining a sufficient resource supply for other species. Under these conditions, the affected species will eventually go extinct for high mortality rates, but this will not affect the other species.

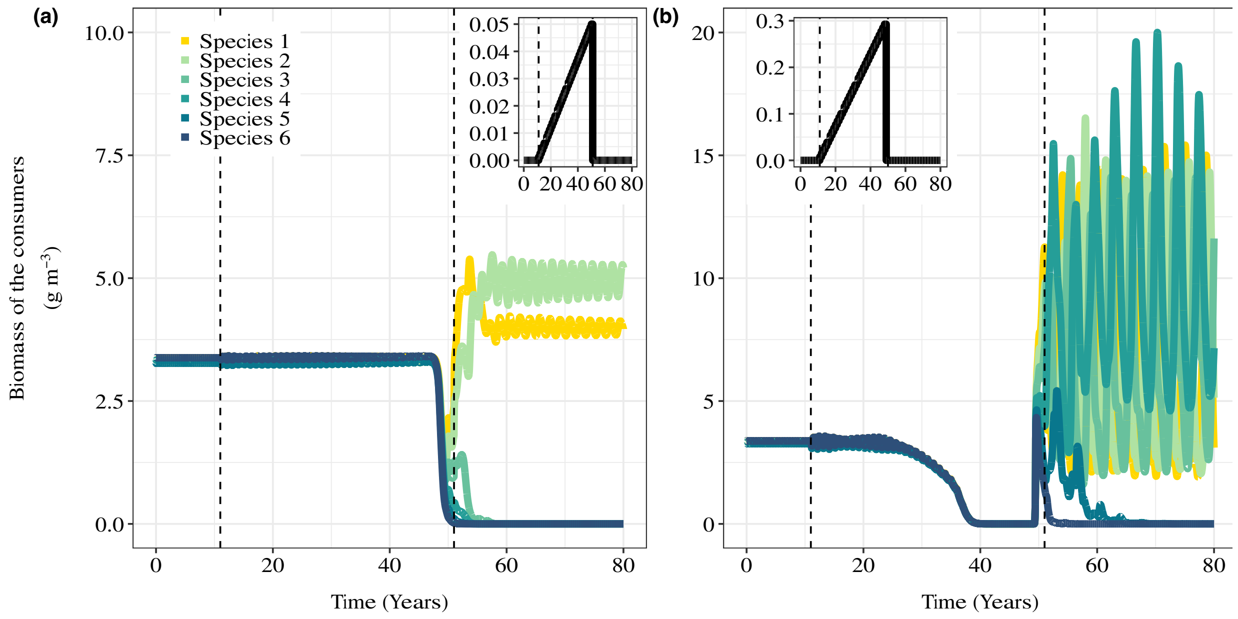

Figure S4: In both panels, mortality for small individuals of all species is increased ($\mu_{\text{min}} = m_{\text{b}}$, $\mu_{\text{max}} = m_{\text{shift}}$). The productivity of the shared resource equals 0.5 g m^{-3} day^{-1} in panel a, and 1.5 g m^{-3} day^{-1} in panel b. The insets in each panel show the change in the focal parameter, the vertical dashed lines indicate where the focal parameter starts to increase and when it is set back to its original value. Note that in panel b, the collapse happens only for high mortality rates, and most species will be able to re-invade after the size-specific mortality is set back to 0. Other parameters have default values.

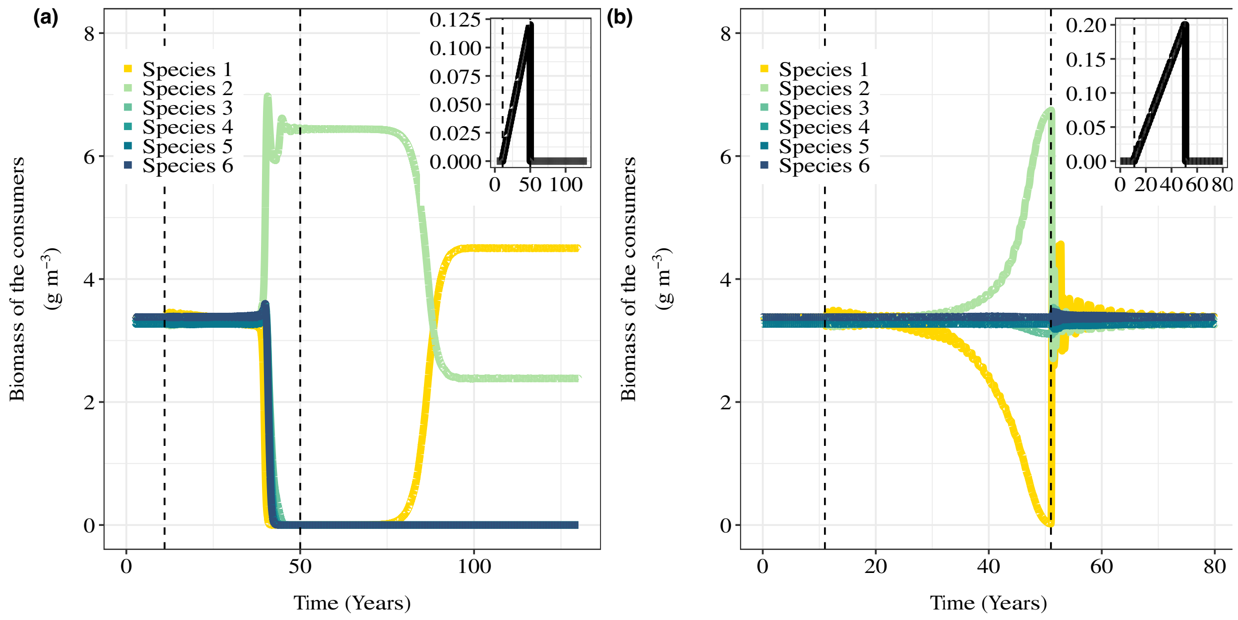

Figure S5: In both panels, mortality for small individuals of species 1 is increased ($\mu_{\text{min}} = m_{\text{b}},\ \mu_{\text{max}} = m_{\text{shift}}$). The productivity of the shared resource equals 0.4 g m^{-3} day^{-1} in panel a, and 0.5 g m^{-3} day^{-1} in panel b. The insets in each panel show the change in the focal parameter, the vertical dashed lines indicate where the focal parameter starts to increase and when it is set back to its original value. Other parameters have default values.
